# Amino acid pattern reveals multi-functionality of ORF3 protein from HEV

**DOI:** 10.6026/973206300200121

**Published:** 2024-02-29

**Authors:** Zoya Shafat, Asimul Islam, Shama Parveen

**Affiliations:** 1 Centre for Interdisciplinary Research in Basic Sciences, Jamia Millia Islamia, New Delhi, India

**Keywords:** Hepatitis E virus (HEV), Open reading frame 3 (ORF3), amino acid composition, structural analysis, Disorder variant, moderately disordered protein, highly disordered protein, Intrinsically Disordered Protein Region (IDPR), Intrinsically Disordered Protein (IDP)

## Abstract

The smallest open reading frame (ORF) encoded protein ORF3 of hepatitis E virus (HEV), recently, has been demonstrated to perform multiple functions besides accessory roles. ORF3 could act as a target for vaccine against HEV infections. The IDR (intrinsically disordered region); IDP (ID protein)/IDPR (ID protein region), plays critical role in various regulatory functions of viruses. The dark proteome of HEV-ORF3 protein including its structure and function was systematically examined by computer predictors to explicate its role in viral pathogenesis and drug resistance beyond its functions as accessory viral protein. Amino acid distribution showed ORF3 enrichment with disorder-promoting residues (Ala, Pro, Ser, Gly) while deficiency in order-promoting residues (Asn, Ile, Phe, Tyr and Trp). Initial investigation revealed ORF3 as IDP (entirely disordered protein) or IDPR (proteins consisting of IDRs with structured globular domains). Structural examination revealed preponderance of disordered regions interpreting ORF3 as moderately/highly disordered protein. Further disorder predictors categorized ORF3 as highly disordered protein/IDP. Identified sites and associated-crucial molecular functions revealed ORF3 involvement in diverse biological processes, substantiating them as targets of regulation. As ORF3 functions are yet to completely explored, thus, data on its disorderness could help in elucidating its disorder related functions.

## Background:

Hepatitis E virus (HEV), of the Hepeviridae family, is a major zoonotic pathogen causing acute hepatitis E worldwide [1]. Recent data has roughly calculated that about 939 million of the world population has been already exposed with HEV infection (past experience) and about 15 - 110 million individuals in the world are experiencing HEV infection (recently experiencing) [2]. In India, the Hepatitis cases reported in India to the Central Bureau of Health Intelligence (CBHI) is exceedingly low, as most of the cases reach to traditional healers for the fact that there is no cure in allopathy as a common belief. Moreover, due to inadequate information, the exact number of HEV cases in our country has been unrecognizable. However, available reports have suggested that HEV is responsible for both acute hepatitis (10-40%) as well as liver failure (15-45%) in India [3, 4]. Currently, HEV constitutes 8 genotypes (GTs) (GT I - GT VIII). The GTs (I and II) infect humans and majorly transmission occurs through spoiled or infected water and are cause acute hepatitis. The GTs (III and IV) constitutes an extended host range [5,6,7] and cause chronic hepatitis (recipients with organ transplantation) [8,9]. Some other HEV strains have been identified from specific hosts, for instance, GTs (V and VI) from wild boars [10, 11] and GT VII and GT VIII from camels [12, 13]. Utilization of improperly cooked meat (from animal) products is one of the chief causes of sporadic cases in developed nations [14]. The HEV expanding host range and newly discovered strains further complicates its implications on human health, its transmission and risk of infection [14]. Also, blood-mediated [15] as well as person-to-person [16] transmission have been reported in addition to transmission from pet animals to humans [17, 18]. Due to all this, HEV has attained global attention and is recognized as a major health burden. Anti-HEV antibodies IgG and IgM, serve as markers for individuals who have experienced past HEV infection (persisting for various years) and person who has ongoing infection (persists for few months) respectively [19, 20]. The three well-defined open reading frames (ORFs), i.e., ORF1, ORF3 and ORF3 forms the genome of HEV [21]. The largest reading frame ORF1 codes for several non-structural proteins that are required for the replication of HEV [22,23]. The translation product of the structural reading frame ORF2 forms the virion major component, i.e., viral capsid [24, 25], and the third reading frame ORF3 at 3' terminus codes for a protein that serves regulatory functions [26,27,28]. Here, current study has shown the analysis on unknown (in terms of structure) regions (i.e., a proteome's fraction which has no noticeable resemblance to some PDB structure) of the ORF3 protein of HEV. This fraction of proteome is considered as the 'dark proteome'. The dark proteomes include the complete proteome with particular emphasis on intrinsically disordered regions (IDRs), i.e., intrinsically disordered protein region (IDPR)/intrinsically disordered protein (IDP), that lack definite (three-dimensional) structures within viral proteomes [29]. Studies have shown the correlation of viral disordered protein segments with its pathogenesis [30,31]. In addition to this, reports have also documented the association of IDPs with several diseases' as they perform diverse roles in regulatory processes. Due to IDP's involvement in important biological processes, these are considered as potential drug targets [32, 33,34,35]. Although, initially ORF3 was just considered a protein having accessory roles; but recently its functions have been associated to biogenesis of quasi-enveloped viral particles; cellular signalling and regulation of immune response and host tropism of HEV. Additionally, its potential to act as vaccine against HEV has also been documented [36,37]. In this context, we conducted computational analysis of the HEV ORF3 proteins through analyzing its intrinsically disordered regions to gain advances in its function via disordered regions. The intrinsic disorderness in the HEV ORF3 was scrutinized using computational approach to envisage its disorder-related functions. The disorder analysis results predicted ORF3 protein highly disordered, which was found to be associated to several important molecular functions and biological processes like binding sites (such as, ion-, protein-, metal-binding), viral replication and RNA biosynthetic process), in addition to occurrence of post-translationally modified sites in its polypeptide chain. On summing up these observations, our study clearly indicated the ORF3 protein involvement in various significant processes as well as its interaction with the membrane of the host cell. The presented study can provide some novel insights into the understanding of ORF3 protein functions besides its accessory roles in HEV life cycle.

## Materials and Methods:

## Sequence retrieval:

The sequences of HEV ORF3 protein were procured from GenBank, housed in NCBI (National Center for Biotechnology Information). The obtained sequences encompassed different GTs GT II, GT III, GT IV, GT V, GT VI, GT VII and GT VIII) and hosts (Human, Wild boar, Swine and Camel), as mentioned in Table 1.

## Amino acid composition prediction:

The amino acid distribution pattern in HEV ORF3 was examined through an online server Expasy ProtParam [38]. The tool ProtParam allows computation of various parameters for the entered protein sequence provided by a user.

## Three dimensional (3D) structure analyses with disorder prediction:

The 3D models of HEV ORF3 protein were predicted using I-TASSER [39] webserver and analyzed. The ORF3 structures were constructed through I-TASSER using threading-based approach. Additionally, we measured the secondary structure content in the ORF3 models using Phyre2 (Protein Homology/AnalogY Recognition Engine) [40] webserver.

Further, the occurrence of the intrinsic disorder within HEV ORF3 proteins was predicted using PONDR (Predictor of Natural Disordered Regions) [41], an online tool, at its default settings. The different versions of PONDR including VSL2, VL3 and VL-XT, were used to evaluate the intrinsic disorder status of the ORF3 proteins.

## Potential disorder-based binding site prediction:

The disorder-based protein binding residues of the ORF3 proteins were identified using a combination of two webservers DISOPRED3 [42] and IUPred2A [43]. The 0.5 was used as the cut off score for the disordered-protein binding residue prediction for both webservers, i.e., DISOPRED3 and IUPred2A.

## Phosphorylation prediction:

The residues that can be phosphorylated, such as, Ser, Tyr and Thr, were identified within the ORF3 proteins of HEV using DEPP (Disorder enhanced phosphorylation prediction) online tool.

## Structure-based function prediction:

The possible gene ontology based-function and process, using obtained HEV ORF3 3D modelled structures, was explored using COFACTOR algorithm [39].

## Results:

**Figure 1:**
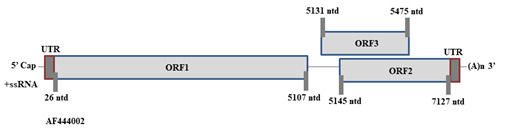
Illustration depicting HEV genome. The genome is systematically organized into 3 ORFs, i.e., ORF1, ORF3 and ORF3. The nucleotide positions of the ORFs in HEV genome is with reference to Sar55 strain (having accession ID AF444002) [44].

The HEV genome encodes 3 well-defined ORFs, i.e., ORF1, ORF2 and ORF3. The ORF3 starts at 5131st nucleotide position while terminates at 5475th nucleotide position. The HEV genome diagrammatic illustration, according to the GenBank Accession ID: AF444002 is shown in Figure 1 [44].

## Evaluation of amino acid patterns:

**Figure 2:**
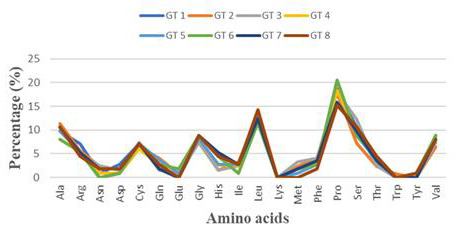
Amino acid distribution pattern analysis in HEV-ORF3. The amino acids percentage in ORF3 sequences was computed using Protparam tool. The sequences include GT I (JF443720), GT II (M74506), GT III (AB222182), GT IV (GU119961), GT V (AB573435), GT VI (AB602441), GT VII (KJ496143) and GT VIII (KX387865).

The evaluation of amino acid patterns in ORF3 polypeptide sequences was carried out to reveal distinctive features of the ORF3. The computed percentage of amino acids in ORF3 is stated in Table 1. Our analysis revealed that ORF3 polypeptides were deficient in most of the order-promoting residues which included Asn, Ile, Phe, Trp and Tyr, while showed normal fractions of Cys, however, the ORF3 proteins were richly endowed with order-promoting residues, such as, Leu and Val. On the contrary, abundance of most of the disorder-promoting residues, such as, Ala, Gly, Pro and Ser were observed in the ORF3 protein sequences, with normal percentage of Arg. In addition to this, the other disorder-promoting residues, like, Gln and Glu were observed in negligible amounts and Lys was found to be absent in the ORF3 protein's polypeptide (Figure 2). The major amino acids that contributed to the ORF3 polypeptide chains included Pro, Leu, Ser, Ala, Gly and Val, which clearly revealed the abundance of disorder-promoting residues (Pro, Ser, Gly and Ala) with limited number of order-promoting residues (Leu and Val). It is noteworthy to mention that the most represented amino acid in ORF3 polypeptide chain was Pro which is a disorder-promoting amino acid (Figure 2). On summing up these observations, our initial analysis interpreted ORF3 proteins either as IDP (entirely disordered protein) or IDPR (proteins consisting of intrinsically disordered regions in combination with structured globular domains) [29]. Therefore, in this regard, our composition analysis further prompted us to evaluate the disorder distribution in the ORF3 polypeptide chains through different bioinformatics predictors.

## Disorder in ORF3 polypeptide chains:

## Quantifying disorder by calculating the predicted percentage of disordered residues

We classified the HEV ORF3 into; structured proteins, moderately disordered proteins and highly disordered proteins based on their overall fraction of predicted intrinsic disorder, i.e., <10% disorder, ≥10-<30% disorder and ≥30% disorder, respectively [45]. Further, we categorically grouped the ORF3 proteins into; ORDPs, IDPRs and IDPs based on the overall fraction of disordered residue and length of disordered domain [46].

## (i) ORDPs (ordered proteins):

These proteins consist of disordered residues less than 30% in their polypeptide chains and are characterized by lack of disordered domain at either C- terminus or N-terminus (disordered segment of 30 or more consecutive amino acid residue); or in positions distinct from terminals N- and C (disordered segment of 40 or more consecutive amino acid residue).

## (ii) IDPRs (structured proteins with IDRs):

These proteins consist of disordered residues less than 30% in their polypeptide chains, however, they are characterized by atleast one disordered domain either at C- terminus or N-terminus (disordered segment of 30 or more consecutive amino acid residue); or in positions distinct from terminals N- and C (disordered segment of 40 or more consecutive amino acid residue).

## (iii) IDPs (intrinsically disordered/unstructured proteins):

These proteins consist of disordered residues more than 30% in their polypeptide chains.

## 3D modelled structures with predicted disorder:

**Figure 3:**
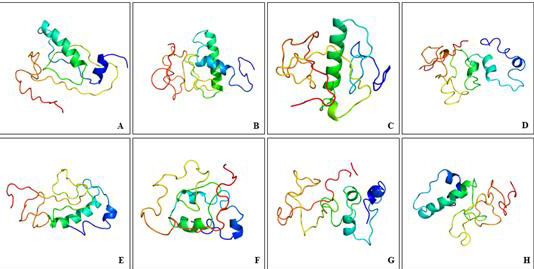
Generated homology modelled 3D structures of HEV-ORF3. (A) GT I (JF443720), (B) GT II (M74506), (C) GT III (AB222182), (D) GT IV (GU119961), (E) GT V (AB573435), (F) GT VI (AB602441), (G) GT VII (KJ496143) and (H) GT VIII (KX387865). The 3D models were generated using Phyre2 webserver.

Figure 2 provides 3D depictions of the ORF3 proteins, generated through I-TASSER, from various HEV viruses. The two major secondary structures in form of alpha-helices and beta strands in combination with disordered regions were identified in modelled ORF3 structures as summarized in Table 2 (Figure 3).

The 3D structures showed the dominance of loops or coils as disordered segments are necessarily present within loops/coils in proteins [42]. As mentioned in Table 2, the identified disorder percentage in generated ORF3 modelled structures clearly indicated the significant amount of intrinsic disorder in ORF3 proteins. The disorder prediction through Phyre2 modelled structures revealed ORF3 as moderately disordered proteins (≥10 - <30% disorder) or highly disordered proteins (≥30% disorder) on the basis of overall predicted intrinsic disorder fraction. Further, the analysis ruled out the probability of ORF3 protein categorization into highly ordered proteins as it was characterized with absence of less than 10% of the disordered segments in its polypeptide chain (highly ordered proteins PPID <10%). Therefore, the presence of significant fraction of disorder in ORF3 proteins, prompted us further to evaluate its disorderness using different PONDR algorithms, i.e., VSL2, VL3 and VL-XT.

##  Disorder analysis with PONDR-VLXT, PONDR-VL3 and PONDR-VSL2:

The predisposition for intrinsic disorder in HEV ORF3 proteins was evaluated using PONDR. Scores > 0.5 corresponded to disordered residues, wherein, different colours were used to depict the disordered regions in ORF3 proteins. The areas in purple are the predicted disordered protein regions by PONDR-VSL2, the regions marked with blue are disordered protein regions by PONDR-VL3 while the regions indicated with red were predicted to be disordered by PONDR-VLXT.

**Figure 4:**
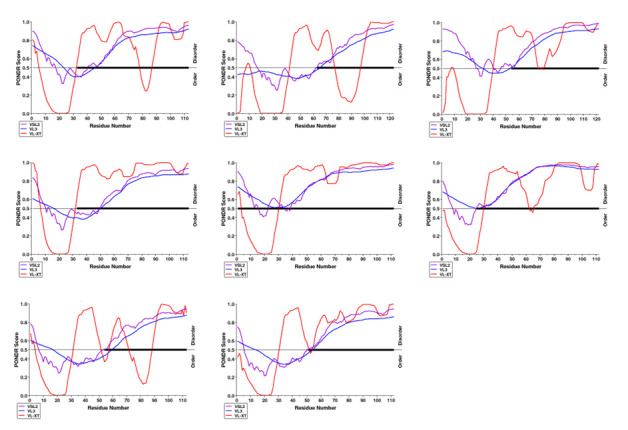
Intrinsic disorder analysis of HEV-ORF3. Intrinsic disorder distribution patterns depicted by graphs (A-H), (A) GT I (JF443720), (B) GT II (M74506), (C) GT III (AB222182), (D) GT IV (GU119961), (E) GT V (AB573435), (F) GT VI (AB602441), (G) GT VII (KJ496143) and (H) GT VIII (KX387865). The analysis was conducted through PONDR (VSL2, VL3 and VL-XT). Disorder probability was computed by setting 0.5 threshold values (dashed line). The regions above this threshold value are estimated as disordered.

The predicted disorder patterns of ORF3 polypeptides, obtained from disorder predictors, are mentioned in Table 3. The disorder distribution profiles of the ORF3 proteins are shown in Figure 4A - H.

## ORF3 protein (JF443720):

The ORF3 polypeptide JF443720 was revealed as a highly disordered protein as it consisted of >30% of disordered residues (68.14% by VLXT, 80.53% by VL3 and 79.65% by VSL2). Additionally, presence of disordered domain in ORF3 polypeptide at the C-terminus, i.e., upto 48 to 73 consecutive amino acid residues, grouped it into IDP (as computed by all PONDR members).

## ORF3 protein (M74506):

The ORF3 polypeptide M74506 was revealed as a highly disordered protein as it consisted of >30% of disordered residues (52.03% by VLXT, 47.15% by VL3 and 62.60% by VSL2). Additionally, disordered domain in ORF3 polypeptide at C-terminus, i.e., upto 35 to 61 consecutive amino acid residues, grouped it into IDP (as computed by all PONDR members).

## ORF3 protein (AB222182):

The ORF3 polypeptide AB222182 was revealed as a highly disordered protein as it consisted of >30% of disordered residues (66.39% by VLXT and 88.52% by VSL2). Additionally, presence of disordered domain in ORF3 polypeptide at the C-terminus, i.e., upto 43 to 66 consecutive amino acid residues, grouped it into IDP (as computed by two PONDR members: VLXT and VSL2).

## ORF3 protein (GU119961):

The ORF3 polypeptide GU119961 was revealed as a highly disordered protein as it consisted of >30% of disordered residues (77.19% by VLXT, 70.18% by VL3 and 67.54% by VSL2). Additionally, disordered domain in ORF3 polypeptide at the C-terminus, i.e., upto 82 to 64 consecutive amino acid residues, grouped it into IDP (as computed by all PONDR members).

## ORF3 protein (AB573435):

The ORF3 polypeptide AB573435 was revealed as a highly disordered protein as it consisted of >30% of disordered residues (75.89% by VLXT, 100.00% by VL3 and 91.07% by VSL2). Additionally, presence of disordered domain in ORF3 polypeptide at the C-terminus, i.e., upto 74 to 112 consecutive amino acid residues, grouped it into IDP (as computed by all PONDR members).

## ORF3 protein (AB602441):

The ORF3 polypeptide AB602441 was revealed as a highly disordered protein as it consisted of >30% of disordered residues (70.54% by VLXT, 48.21% by VL3 and 85.71% by VSL2). Additionally, presence of disordered domain in ORF3 polypeptide at the C-terminus, i.e., upto 47 to 88 consecutive amino acid residues, grouped it into IDP (as computed by all PONDR members).

## ORF3 protein (KJ496143):

The ORF3 polypeptide KJ496143 was revealed as a highly disordered protein as it consisted of >30% of disordered residues (55.75% by VLXT, 58.41% by VL3 and 58.41% by VSL2). Additionally, presence of disordered domain in ORF3 polypeptide at the C-terminus, i.e., upto 25 to 60 consecutive amino acid residues, grouped it into IDP (as computed by all PONDR members).

## ORF3 protein (KX387865):

The ORF3 polypeptide KX387865 was revealed as a highly disordered protein as it consisted of >30% of disordered residues (70.54% by VLXT, 63.39% by VL3 and 59.82% by VSL2). Additionally, presence of disordered domain in ORF3 polypeptide at the C-terminus, i.e., upto 58 to 62 consecutive amino acid residues, grouped it into IDP (as computed by all PONDR members).

## Categorizing ORF3 protein into disorder variant:

To make our findings more transparent, the results were combined (obtained from different disorder predictors) that revealed HEV ORF3 a highly disordered protein as the overall intrinsic disorder fraction was predicted to be ≥30% in the polypeptide) or IDP (as the predicted overall percentage of disordered residues was >30% in combination with disordered domain in the polypeptide) as mentioned in Table 3. Thus, huge content of intrinsic disorder in the HEV-ORF3 protein signified its interacting ability with other molecules by revealing its disorder-based binding tendency. Moreover, the presence of disordered domains at the C-terminus of ORF3 protein showed its propensity of binding to the ORF2 protein as well as the host components. As our intrinsic disorder propensity analysis is in line with the initial disorder prediction, thus, we further examined the protein-binding regions in the ORF3 proteins to make our findings more elaborative and consistent.

## Potential disorder-based binding protein regions:

**Figure 5:**
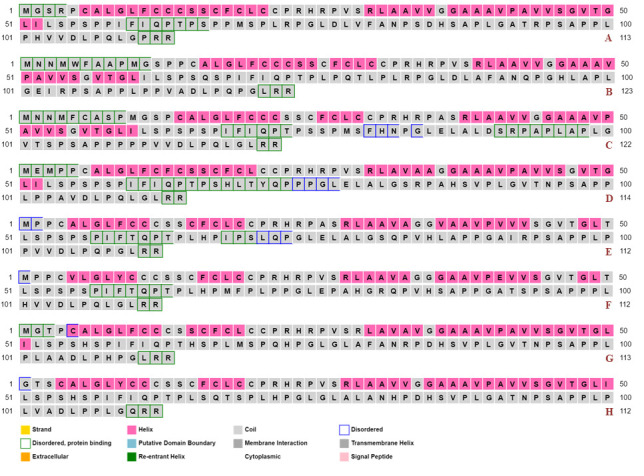
Representation of disordered protein binding residues in HEV-ORF3. The disordered protein binding residues in ORF3 amino acid sequences are represented in green outlined boxes. The major secondary structure elements including alpha-helices and beta-sheets are also depicted. The analysis was conducted using PSIPRED.

The disordered protein binding residues within disordered ORF3 protein sequences predicted by identified and are mentioned in the table (Table 4). The identified disordered protein binding residues using DISOPRED3 is shown in Figure 5. Thus, the identified protein-binding propensity analyses of the HEV-ORF3 are also in line with the initial disorder prediction as protein-binding sites (as predicted by DISOPRED3 and IUPred2A) were predicted towards both N- and C-terminus of the ORF3 protein sequences.

## Evaluation of phosphorylation patterns:

**Figure 6:**
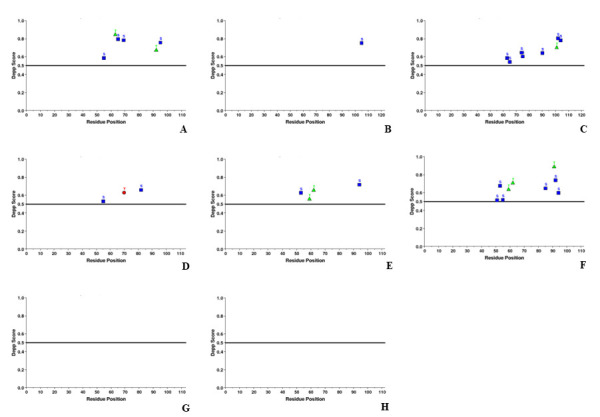
Identification of phosphorylation sites (Serine, Threonine, Tyrosine) within HEV-ORF3, (A) GT 1 (JF443720), (B) GT 2 (M74506), (C) GT 3 (AB222182), (D) GT 4 (GU119961), (E) GT 5 (AB573435), (F) GT 6 (AB602441), (G) GT 7 (KJ496143) and (H) GT 8 (KX387865). The resulting score was computed using DEPP. The line (0.5 threshold value) is set to discriminate ordered and disordered residues. The predicted phosphorylated residues above the threshold are represented as: Ser (S): Blue, Thr (T): Green, and Tyr (Y): Red.

The predicted phosphorylation sites (P-sites) within HEV ORF3 are mentioned in Table 5 (Figure 6). The phosphorylation pattern showed that Ser (rather than Thr and Tyr) was the most represented phosphorylated residue while Tyr was the least represented residue (Figure 6). Moreover, the results showed that most of the P-sites were found to be prevalent in the disordered ORF3 regions (Figure 4).

## Prediction of gene ontology terms through COFACTOR algorithm:

The three top ranked molecular functions and biological processes based on 3D modelled ORF3 structures, generated through I-TASSER, are mentioned and described in Table 6.

The binding functions such as protein binding (GO: 0005515), DNA binding (GO: 0003677), flavin adenine dinucleotide binding (GO: 0050660) were attributed to HEV-ORF3, that showed the tendency of ORF3 protein to bind to varied molecules (Table 6). Furthermore, the involvement of ORF3 protein in positive regulation of transcription (GO: 0045893), glucose catabolic process (GO: 0006007), hexose biosynthetic process (GO: 0019319), carbohydrate metabolic process (GO: 0005975), revealed the significant biological processes attributed to ORF3 (Table 6).

## Discussion:

The ORF3 protein has recently been linked to host immunity and signalling, host tropism and vaccine target [36, 37], henceforth, its targeting is ideal for devising treatment against HEV. In view of this, we performed a sequence-based analysis on the HEV ORF3 sequences to shed light into their intrinsic disorder prevalence by employing bioinformatics approach. This novel study reports the elucidation of ORF3 protein unstructured regions to shed lights on its implications in HEV regulation and pathogenesis. As disordered regions are rooted in the idiosyncrasies of their amino acid composition, we examined the amino acid composition of the ORF3 polypeptides in order to reveal its residue percentages. Investigations have revealed that IDRs (IDPRs/IDPs) possess a peculiar pattern of amino acid sequences, which differentiate them from ordered proteins [48,49,50, 51]. As suggested in reports, the IDRs are enriched with disorder-promoting residues, such as, Ala (A), Arg (R), Gly (G), Gln (Q), Ser(S), Pro (P), Glu (E) and Lys (K), while are deficient in order-promoting residues, such as, Trp (W), Cys (C), Phe (F), Ile (I), Tyr (Y), Val (V), Leu (L) and Asn (N) [48,51]. It was also proposed that His (H), Met (M), Thr (T) and Asp (D) are neither order-promoting amino acids nor disorder-promoting amino acids [48,51]. The topmost contributing amino acids to the ORF3 polypeptides included Pro, Leu, Ser, Ala, Gly and Val residues. These residues involved limited number of order-promoting residues (Leu and Val) and abundance of disorder-promoting residues (Pro, Ser, Gly and Ala). Additionally, Pro, a disorder-promoting residue, was the most represented amino acid constituting the ORF3 polypeptide chains. These results clearly indicated the ORF3 proteins substantial enrichment with disorder-promoting amino acids, revealing ORF3 either as IDPR, i.e., protein consisting of intrinsically disordered regions in combination with structured globular domains or IDP, i.e., entirely disordered protein [29]. Thus, our initial findings predicted the ORF3 proteins with significant intrinsic disorder prevalence. Inclusive scrutinization of protein structures provides knowledge about its functions, in this context, we further scrutinize the ORF3 structures (obtained 3D models) for its intrinsic disorder content. The modelled I-TASSER structures revealed two major forms of secondary structure elements (alpha helices and beta strands) in combination with disordered regions. The predominance of coils in ORF3 protein models was revealed, as it has been suggested that though loops (or coils) are not necessarily disordered, however, the disordered segments in proteins are only found inside loop or coils [47]. The obtained ORF3 modelled structures (generated through Phyre2) was revealed either as moderately disordered proteins or highly disordered proteins based on criterion suggested [45]. Thus, the ORF3 structural analysis was in excellent agreement with our initial amino acid compositional findings suggesting ORF3 proteins with significant percentage of IDRs. The prevalence of IDRs, i.e., IDPR or IDP in ORF3 prompted us to further evaluate its disorder status. The evaluation of disorder patterns in ORF3 polypeptides was carried out using different computational predictors. The PONDR algorithm PONDR-VL3 was chosen as it shows high accuracy over long disordered regions prediction [52], whereas the disorder predictor PONDR-VLXT was chosen because of its very extreme sensitivity [53,54]. PONDR makes prediction upon single amino acid sequence [55]. The HEV ORF3 proteins were categorically differentiated into ORDP, IDPR and IDP [46]. On applying this aforementioned criterion, our disorder profiles, obtained from PONDR disorder predictors, revealed ORF3 as IDPs. The different stages in the life cycle of a virus, such as, attachment, entry, seizing the host machinery, synthesis of viral component and assembly and subsequently exit from host organisms, greatly depend on the occurrence of disorderness in their proteomes [56]. This type of relation, i.e., relation between IDRs and specific roles [57], have been shown in HCV (hepatitis C virus) [58], MeV (Measles virus) [59], Hendra virus [60]. Additionally, it is important to mention that recent HEV reports have shown their regulation mechanism linked to characteristic disorderness possessed by them, for instance, non-structural ORF1 PPR (Polyproline region) domain [61], non-structural ORF1 Y-domain [62], and other proteins [63, 64,65,66, 67,68]. Recent study on ORF2 has also shown the importance of disordered regions in HEV regulation [69]. In this regard, it is important to mention that disordered ORF3 protein regions could perform critical regulatory functions via interaction with host and viral components. Our disorder prediction showed that out of the N- and C-terminals, the C-terminal showed significant disorderness as compared to the initial N-terminus. Sequence analysis studies on HEV-ORF3 have shown that the N-terminal region (of about 25 aa) is conserved in all eight GTs in comparison to the other regions of ORF3 protein [70,71], which perhaps reflects the conserved virion release role associated with ORF3 protein [72]. Further, the C-terminus of ORF3 is less conserved in HEV GTs, particularly from 62 to 114 aa, thus this specific region is responsible for providing adaptation in different hosts. Moreover, it has been suggested that host-specific pattern exists for ORF3 that may influences the host tropism [73,74] and genotype-specific evolution patterns influence the ORF3 protein functions [75]. The significance of disorder proteins has also been implicated in a variety of binding functions, such as, protein binding [48, 76]. Reports have demonstrated the involvement of MoRFs in viruses' life cycles [77,78,79]. The MoRF is termed as a short segment within disordered protein segments (IDPR/IDP) that undergoes disorder-to-order state transition upon binding to its partner [80]. Herein, the MoRFs were predicted in ORF3 proteins by two predictors (DISOPRED3 and IUPred2A). The server DISOPRED3 identifies the protein binding disordered regions within a given sequence target [81]. This study chosen DISOPRED3 (over DISOPRED2) for IDRs identification as it provides substantially improved results [81]. In addition to this, IUPred2A was employed to examine the binding regions within disordered ORF3 protein segments [82]. IUPred3 and IUPred2A allow identification of both disordered protein regions (through IUPred3/IUPred2) and disordered binding regions (through ANCHOR2) [82,83]. It is remarkable to state that the maximum number of identified protein-binding residues in the ORF3 protein sequences also showed propensity towards the C-terminus. Thus, these hypotheses substantiate our present findings. Further, we predicted the phosphorylated residues in ORF3 protein sequences as reports have revealed the importance of post-translational modifications (PTMs) in numerous processes (protein folding, signal transduction, apoptosis, etc) [84], as well as in the infection cycle of intracellular pathogens [85,86], like Alphaviruses [87, 88] and Flaviviruses [89,90, 91]. Our phosphorylation patterns of ORF3 protein sequences showed P-sites at their C-terminals, in which, the P-sites showed prevalence within disordered segments of the ORF3 polypeptides that inferred strong correlation between phosphorylation and disorder ORF3 regions as reported earlier [92,93]. As suggested, disordered segment of protein regions displays sites for PTM perhaps due to flexibility (conformational) of display sites provided by the disordered regions in the proteins [94,95]. Report demonstrates that Serine's hydroxyl group act as targets (by kinase proteins) for phosphorylation, within disordered protein segments [96]. Consequently, higher predicted number of phosphorylated Serine residues in ORF3 protein revealed its interaction ability and flexible tendency, eventually, relating its importance in protein regulation. The obtained results from this study are in accordance with the previous investigations on ORF3 protein revealing its role in virus cell interaction [97], modulation of multiple signaling pathways, (includes pathways of host innate immunity) and subsequently virus pathogenesis [98,99]. This substantiates our present hypothesis which suggests the involvement of ORF3 in regulation and pathogenesis of HEV through its order/disorder tendency. Furthermore, we carried out the prediction of 3D structured models of ORF3 protein. Using the predicted models the different molecular function and biological process was determined [41,42]. Several functions including protein binding, DNA binding, flavin adenine dinucleotide binding, were predicted which clearly uncovered ORF's propensity to bind to several types of molecules, which have been previously reported in regulation [100]. It is interesting to mention that the involvement of ORF3 in significant processes, such as, axon guidance [101], and in regulation of neuron apoptotic process [102]. This revealed its role in neural development. Axon pathfinding or axon guidance refers to a process by which a neuron sends out axons to reach their correct targets. Study has demonstrated the role of the axon guidance signalling pathways in gene expression control [103]. Neuronal apoptotic cell death regulation process plays a major role in shaping the nervous system development during embryogenesis [104]. Furthermore, the identified processes, for instance, exocytosis, proteolysis, acute inflammation, transcription regulation and cell wall organization, further signified the critical role played by ORF3 in HEV regulation and pathogenesis. Altogether, the ORF3-associated molecular functions and biological processes clearly showed its involvement in HEV in multiple crucial roles [43]. Importantly, IDPR/IDP has been associated with the regulation of as well as interaction with multiple unrelated partners due to its complex and heterogeneous structural organization, thus, constituting it as a multifunctional molecule [105]. Thus, these observations further substantiate our findings. Altogether, our findings from the current study hypothesized ORF3 as a protein associated with multiple functions beyond its accessory roles in HEV.

## Conclusions:

The study sheds novel light on the extent of intrinsic disorder distribution in the ORF3 protein of HEV. The sequences were utilized from the publicly available online database to perform comprehensive computational analysis of the ORF3 by analyzing the extent of occurrence of intrinsic disorder in HEV. The ORF3 protein sequences revealed abundance of signature disorder-promoting amino acid residues, which clearly indicated the ORF3 protein either as IDPR, i.e., protein consisting of intrinsically disordered regions in combination with structured globular domains or IDP, i.e., entirely disordered protein. Generated modelled ORF3 structures revealed the presence of significant fraction of disorder interpreting it as moderately disordered/highly disordered variant. Our predicted structural analysis was in accordance with initial amino acid compositional analysis which suggested ORF3 with significant percentage of IDRs. The prevalence of IDRs (IDPRs/IDPs) in ORF3 further urged us to evaluate its disorder status. The examination of disorder distribution (through different predictors) categorized ORF3 as IDP or highly disordered proteins, thus suggesting its involvement in various significant regulatory functions of viruses. It was observed that C-terminus had larger fraction of intrinsic disorder than the N-terminus. Additionally, the identified maximum number of protein-binding residues in the ORF3 protein sequences also showed propensity towards the C-terminus. The presence of post-translational modifications (like phosphorylation) in ORF3 protein further signified its involvement in various important mechanisms. Subsequently, identified structure-based gene ontology terms clearly revealed multiple functions associated with ORF3. Our study in near future may provide critical information on the unknown functions associated with the HEV-ORF3 protein.

## Data Availability:

The sequences accession ID numbers are available in GenBank Overview (nih.gov)

## Funding:

The author(s) received no financial support for the research, authorship, and/or publication of this article.

## Authors' contributions:

SP conceptualized the research. ZS was a major contributor in writing the manuscript and performed the biocomputational analysis of the protein. AI and SP proofread the manuscript. All the authors read and approved the final manuscript.

## Figures and Tables

**Table 1:** Amino acid distribution pattern prediction in HEV-ORF3 sequences

**AA**	**GT I**	**GT II**	**GT III**	**GT IV**	**GT V**	**GT VI**	**GT VII**	**GT VIII**
*Ala*	9.7	11.4	10.7	10.5	9.8	8	10.6	10.7
*Arg*	7.1	5.7	4.9	5.3	5.4	5.4	5.3	4.5
*Asn*	0.9	2.4	2.5	0.9	-	-	1.8	1.8
*Asp*	2.7	1.6	1.6	0.9	0.9	0.9	1.8	1.8
*Cys*	7.1	6.5	7.4	6.1	7.1	7.1	7.1	7.1
*Gln*	1.8	4.1	1.6	2.6	3.6	2.7	1.8	2.7
*Glu*	-	0.8	0.8	1.8	0.9	1.8	-	-
*Gly*	8	8.1	7.4	7.9	8	8.9	8.8	8.9
*His*	2.7	1.6	1.6	2.6	2.7	4.5	5.3	4.5
*Ile*	2.7	3.3	2.5	2.6	2.7	0.9	2.7	2.7
*Leu*	11.5	13	11.5	13.2	13.4	11.6	12.4	14.3
*Lys*	-	-	-	-	-	-	-	-
*Met*	1.8	2.4	3.3	1.8	0.9	1.8	1.8	-
*Phe*	3.5	4.1	4.1	3.5	2.7	2.7	3.5	1.8
*Pro*	18.6	17.9	18	18.4	20.5	20.5	15.9	15.2
*Ser*	10.6	7.3	12.3	9.6	8.9	8.9	9.7	10.7
*Thr*	2.7	2.4	2.5	3.5	3.6	4.5	3.5	4.5
*Trp*	-	0.8	-	-	-	-	-	-
*Tyr*	-	-	-	0.9	-	0.9	-	0.9
*Val*	8.8	6.5	7.4	7.9	8.9	8.9	8	8
Note: The amino acid values are mentioned as percentages.
Note: GT I (JF443720);
GT II (M74506);
GT III (AB222182);
GT IV (GU119961);
GT V (AB573435);
GT VI (AB602441);
GT VII (KJ496143);
GT VIII (KX387865).

**Table 2:** Secondary structure and disorder prediction in HEV-ORF3 proteins

**ORF3 Sequence**	**Disordered (%)**	**Alpha helix (%)**	**Beta strand (%)**
GT I	22	27	19%)
GT II	27	22%	18
GT III	43	30	7
GT IV	20	22	18
GT V	32	26	15
GT VI	31	27	12
GT VII	18	23	19
GT VIII	22	24	21
Note: GT I (JF443720);
GT II (M74506);
GT III (AB222182);
GT IV (GU119961);
GT V (AB573435);
GT VI (AB602441);
GT VII (KJ496143);
GT VIII (KX387865).

**Table 3:** Intrinsic disorder score prediction in the HEV-ORF3 proteins.

**Disordered regions**	**Overall disorder percentage**	**Disordered residues number**	**Longest disordered domain**	**Protein disorder variant Category [45, 46]**
JF443720 [113 AA]				
VLXT				
[1-5] MGSRP [33-77] AVVGGAAAVPAVVSGVTGLILSPSPPIFIQPTPSPPMSPLRPGLD [87-113] SAPLGATRPSAPPLPHVVDLPQLGPRR	68.14	77	45	Highly Disordered Protein OR IDP
VL3				
[1-23] MGSRPCALGLFCCCSSCFCLCCP [46-113] SGVTGLILSPSPPIFIQPTPSPPMS PLRPGLDLVFANPSDHSAPLGATRPSAPPLPHVVDLPQLGPRR	80.53	91	68	Highly Disordered Protein OR IDP
VSL2				
[1-17] MGSRPCALGLFCCCSSC [41-113] VPAVVSGVTGLILSPSPPIFIQPTPSPPMSPLRPGLDLVFANPSDHSAPLGATRPSAPPLPHVVDLPQLGPRR	79.65	90	73	Highly Disordered Protein OR IDP
M74506 [123 AA]				
VLXT				
[8-10] APM [42-76] AVVGGAAAVPAVVSGVTGLILSPSQSPIFIQPTPL [98-123] APLGEIRPSAPPLPPVADLPQPGLRR	52.03	64	35	Highly Disordered Protein OR IDP
VL3				
[66-123] QSPIFIQPTPLPQTLPLRPGLDLAFANQPGHLAPLGEIRPSAPPLPPVADLPQPGLRR	47.15	58	58	Highly Disordered Protein OR IDP
VSL2				
[1-16] MNNMWFAAPMGSPPCA [63-123] SPSQSPIFIQPTPLPQTLPLRPGLDLAFANQPGHLAPLGEIRPSAPPLPPVADLPQPGLRR	62.6	77	61	Highly Disordered Protein OR IDP
AB222182 [122 AA]				
VLXT				
[8-9] SP [41-74] AVVGGAAAVPAVVSGVTGLILSPSPSPIFIQPTP [76-77] SP [80-122] HNPGLELALDSRPAPLAPLGVTSPSAPPPPPVVDLPQLGLRR	66.39	81	43	Highly Disordered Protein OR IDP
VL3				
--				
VSL2				
[1-26] MNNMFCASPMGSPCALGLFCCCSSCF [33-40] HRPASRLA [47-54] AAVPAVVS [57-122] TGLILSPSPSPIFIQPTPSSPMSFHNPGLELALDSRPAPLAPLGVTSPSAPPPPPVVDLPQLGLRR	88.52	108	66	Highly Disordered Protein OR IDP
GU119961 [114 AA]				
VLXT				
[1-6] MEMPPC [33-114] VAAGGAAAVPAVVSGVTGLILSPSPSPIFIQPTPSHLTYQPPPGLELALGSRPAHSVPLGVTNPSAPPLPPAVDLPQLGLRR	77.19	88	82	Highly Disordered Protein OR IDP
VL3				
[1-16] MEMPPCALGLFCFCSS [51-114] LILSPSPSPIFIQPTPSHLTYQPPPGLELALGSRPAHSVPLGVTNPSAPPLPPAVDLPQLGLRR	70.18	80	64	Moderately Disordered Protein OR IDPR
VSL2				
[1-10] MEMPPCALGL [12-13] CF [50-114] GLILSPSPSPIFIQPTPSHLTYQPPPGLELALGSRPAHSVPLGVTNPSAPPLPPAVDLPQLGLRR	67.54	70	65	Highly Disordered Protein OR IDP
AB573435 [112 AA]				
VLXT				
[1-3] MPP [31-112] AVAGGVAAVPVVVSGVTGLTLSPSPSPIFTQPTPLHPIPSLQPGLELALG SQPVHLAPPGAIRPSAPPLPPVVDLPQPGLRR	75.89	85	82	Highly Disordered Protein OR IDP
VL3				
[1-112] MPPCALGLFCCCSSCFCLCCPRHRPASRLAAVAGGVAAVPVVVSGVTGLTLSPSPSPIFTQPTPLHPIPSLQPGLELALG SQPVHLAPPGAIRPSAPPLPPVVDLPQPGLRR	100	112	112	Highly Disordered Protein OR IDP
VSL2				
[1-15] MPPCALGLFCCCSSC [23-34] HRPASRLAAVAG [37-37] A [39-112] VPVVVSGVTGLTLSPSPSPIFTQPTPLHPIPSLQPGLELALG SQPVHLAPPGAIRPSAPPLPPVVDLPQPGLRR	91.07	102	74	Highly Disordered Protein OR IDP
AB602441 [112 AA]				
VLXT				
[31-62] AVAGGGAAVPEVVSGVTGLTLSPSPSPIFTQP [66-112] HPMFPLPPGLEPAHGRQPVHSAPPG ATSPSAPPPLHVVDLPQLGLRR	70.54	79	47	Highly Disordered Protein OR IDP
VL3				
[1-24] MPPCVLGLYCCCSSCFCLCCPRHR [27-112] SRLAAVAGGGAAVPEVVSGVTGLTLSPSPSPIFTQPTPLHPMFPLPPGLEPAHGRQPVHSAPPGATSPSAPPPLHVVDLPQLGLRR	98.21	110	86	Highly Disordered Protein OR IDP
VSL2				
[1-8] MPPCVLGL [25-112] PVSRLAAVAGGGAAVPEVVSGVTGLTLSPSPSPIFTQPTPLHPMFPLPPGLEPAHGRQPVHSAPPGATSPSAPPPLHVVDLPQLGLRR	85.71	96	88	Highly Disordered Protein OR IDP
KJ496143 [113 AA]				
VLXT				
[1-4] MGTP [32-51] VAVGGAAAVPAVVSGVTGLI [58-71] PIFIQPTHSPLMSP [89-113] PLGVTNPSAPPLPLAADLPHPGLRR	55.75	63	25	Highly Disordered Protein OR IDP
VL3				
[1-16] MGTPCALGLFCCCSSC [60-113] FIQPTHSPLMSPQHPGLGLAFANRPDHSVPLGVTNPSAPPLPLAADLPHPGLRR	58.41	66	60	Highly Disordered Protein OR IDP
VSL2				
[1-6] MGTPCA [54-113] PSHSPIFIQPTHSPLMSPQHPGLGLAFANRPDHSVPLGVTNPSAPPLPLAADLPHPGLRR	58.41	66	60	Highly Disordered Protein OR IDP
KX387865 [112 AA]				
VLXT				
[31-51] AVVGGAAAVPAVVSGVTGLIL [55-112] HSPIFIQPTPLSQTSPLHPGLGLALANHPDHSVPLGATNPSAPPLPLVADLPPLGQRR	70.54	79	58	Highly Disordered Protein OR IDP
VL3				
[1-13] GTSCALGLYCCCS [55-112] HSPIFIQPTPLSQTSPLHPGLGLALANHPDHSVPLGATNPSAPPLPLVADLPPLGQRR	63.39	71	58	Highly Disordered Protein OR IDP
VSL2				
[1-5] GTSCA [51-112] LSPSHSPIFIQPTPLSQTSPLHPGLGLALANHPDHSVPLGATNPSAPPLPLVADLPPLGQRR	59.82	67	62	Highly Disordered Protein OR IDP

**Table 4:** Protein binding residues identification of the HEV-ORF3

**ORF3 Protein**	**DISOPRED3 (cutoff = ≥ 0.5)**	**IUPRED2A ANCHOR (cutoff = ≥ 0.5)**
JF443720	[1-4] MGSR [60-66] FIQPTPS [111-113] PRR	[99-113] PLPHVVDLPQLGPRR
M74506	[1-11] MNNMWFAAPMG [121-123] LRR	---
AB222182	[1-9] MNNMFCASP [68-73] IFIQPT [91-98] SRPAPLAP [121-122] RR	[115-122] LPQLGLRR
GU119961	[1-5] MEMPP [60-72] IFIQPTPSHLTYQP [113-114] RR	---
AB573435	[57-63] PIFTQPT [68-70] IPS [111-112] RR	---
AB602441	[57-63] PIFTQPT [111-112] RR	[59-112] FTQPTPLHPMFPLPPGLEPAHGRQPVHSA PPGATSPSAPPPLHVVDLPQLGLRR
KJ496143	[1-3] MGT [111-113] LRR	---
KX387865	[110-112] QRR	---

**Table 5:** Phosphorylated residues identification in HEV-ORF3 proteins

**Sequences**	**Number of phosphorylated residues**		
	**Ser**	**Thr**	**Tyr**
JF443720	4 out 12 (33.33%)	2 out of 3 (66.66%)	0 out of 0 (00.00%)
M74506	1 out 9 (11.11%)	0 out of 3 (00.00%)	0 out of 0 (00.00%)
AB222182	7 out of 15 (46.66%)	1 out of 3 (33.33%)	0 out of 0 (00.00%)
GU119961	2 out of 11 (18.18%)	0 out of 4 (00.00%)	1 out of 1 (100.00%)
AB573435	2 out of 10 (20.00%)	2 out of 4 (50.00%)	0 out of 0 (00.00%)
AB602441	6 out of 10 (60.00%)	3 out of 5 (60.00%)	0 out of 1 (00.00%)
KJ496143	0 out of 11 (00.00%)	0 out of 4 (00.00%)	0 out of 0 (00.00%)
KX387865	0 out of 12 (00.00%)	0 out of 5 (00.00%)	0 out of 1 (00.00%)

**Table 6:** GO term prediction for HEV-ORF3 modelled structure

**GO terms**	**Description**
JF443720	
Molecular Function	
GO: 0050525	Cutinase activity
GO: 0052689	Carboxylic ester hydrolase activity
GO: 0050290	Sphingomyelin phosphodiesterase D activity
Biological Process	
GO: 0006629	Lipid metabolic process
GO: 0019835	Cytolysis
GO: 0044179	Hemolysis in another organism
M74506	
Molecular Function	
GO: 0038023	Signalling receptor activity
GO: 0005080	Protein kinase C binding
GO: 0005520	Insulin-like growth factor binding
Biological Process	
GO: 0001775	Cell activation
GO: 0006887	Exocytosis
GO: 0007411	Axon guidance
AB222182	
Molecular Function	
GO: 0016740	Transferase activity
Biological Process	
None was predicted	
GU119961	
Molecular Function	
GO: 0004650	Poly galacturonase activity
GO: 0005515	Protein binding.
Biological Process	
GO: 0044238	Primary metabolic process
GO: 0071555	Cell wall organization
GO: 0045229	External encapsulating structure organization
AB573435	
Molecular Function	
GO: 0004175	Endo-peptidase activity
Biological Process	
GO: 0002526	Acute inflammatory response
GO: 0043523	Regulation of neuron apoptotic process
GO: 0051094	Positive regulation of developmental process
AB602441	
Molecular Function	
GO: 0050660	Flavin adenine dinucleotide binding
GO: 0003995	Acyl-CoA dehydrogenase activity
GO: 0003677	DNA binding
Biological Process	
GO: 0006508	Proteolysis
GO: 0006635	Fatty acid beta-oxidation
GO: 0045893	Positive regulation of transcription, DNA-templated
KJ496143	
Molecular Function	
GO: 0016832	Aldehyde-lyase activity
Biological Process	
GO: 0006007	Glucose catabolic process
GO: 0006091	Generation of precursor metabolites and energy
GO: 0019319	Hexose biosynthetic process
KX387865	
Molecular Function	
GO: 0004650	Polygalacturonase activity
GO: 0005515	Protein binding
Biological Process	
GO: 0071555	Cell wall organization
GO: 0005975	Carbohydrate metabolic process
GO: 0016226	Iron-sulfur cluster assembly
